# Case-Control Approach to Identify *Plasmodium falciparum* Polymorphisms Associated with Severe Malaria

**DOI:** 10.1371/journal.pone.0005454

**Published:** 2009-05-06

**Authors:** Watcharee Chokejindachai, David J. Conway

**Affiliations:** 1 Department of Infectious and Tropical Diseases, London School of Hygiene and Tropical Medicine, London, United Kingdom; 2 Department of Tropical Pediatrics, Faculty of Medicine, Mahidol University, Bangkok, Thailand; 3 Medical Research Council Laboratories, Fajara, Banjul, The Gambia; University of California Los Angeles, United States of America

## Abstract

**Background:**

Studies to identify phenotypically-associated polymorphisms in the *Plasmodium falciparum* 23 Mb genome will require a dense array of marker loci. It was considered promising to undertake initial allelic association studies to prospect for virulence polymorphisms in Thailand, as the low endemicity would allow higher levels of linkage disequilibrium (LD) than would exist in more highly endemic areas.

**Methodology/Principal Findings:**

Assessment of LD was first made with 11 microsatellite loci widely dispersed in the parasite genome, and 16 microsatellite loci covering a ∼140 kb region of chromosome 2 (an arbitrarily representative non-telomeric part of the genome), in a sample of 100 *P. falciparum* isolates. The dispersed loci showed minimal LD (Index of Association, *I^S^_A_* = 0.013, P = 0.10), while those on chromosome 2 showed significant LD values mostly between loci <5 kb apart. A disease association study was then performed comparing parasites in 113 severe malaria cases and 245 mild malaria controls. Genotyping was performed on almost all polymorphisms in the binding domains of three erythrocyte binding antigens (*eba175*, *eba140* and *eba181*), and repeat sequence polymorphisms ∼2 kb apart in each of three reticulocyte binding homologues (*Rh1*, *Rh2a/b*, and *Rh4*). Differences between cases and controls were seen for (i) codons 388-90 in *eba175*, and (ii) a repeat sequence centred on *Rh1* codon 667.

**Conclusions/Significance:**

Allelic association studies on *P. falciparum* require dense genotypic markers, even in a population of only moderate endemicity that has more extensive LD than highly endemic populations. Disease-associated polymorphisms in the *eba175* and *Rh1* genes encode differences in the middle of previously characterised erythrocyte binding domains, marking these for further investigation.

## Introduction

A wide and unexplained spectrum of clinical disease caused by the malaria parasite *Plasmodium falciparum* is responsible for approximately one million deaths each year, mostly in African children in highly endemic populations [1] but also in adults in areas of lower endemicity [2]. Experimental studies have elegantly demonstrated polymorphic virulence traits within rodent malaria parasite species [3]–[5], and such virulence polymorphism is theoretically expected in *P. falciparum* [6]. Genes encoding proteins that interact with host cell receptors and immune responses are candidates as virulence determinants. There are occasional reports of allele frequency differences between severe and mild malaria isolates, for commonly genotyped polymorphic antigen loci [7]–[13], although associations differ among studies and it is possible that studies showing no differences are unreported. Some attention has focused on sequence motifs in variable multi-copy (*var*) genes that determine infected erythrocyte adhesion phenotypes and are clustered near the telomeres of most of the 14 chromosomes and subject to ectopic recombination and re-positioning [14], [15]. The extent of genetic polymorphism throughout the rest of the genome that displays more conventional Mendelian inheritance [16] is now being more effectively surveyed [17]–[19]. Given the imminent potential of large scale genotyping, there is a need to address whether a genome wide allelic association approach is feasible to identify loci in *P. falciparum* which affect the severity of malaria [20].

An important consideration is that a spectrum of *P. falciparum* genetic population structures exist, depending on endemic infection levels that determine the amount of mixing between unrelated male and female gametocytes, and consequently the effective recombination rate [21]. The highest effective recombination rates (and thereby lowest levels of linkage disequilibrium, LD) are in endemic areas of Africa where multiple genotype infections are common, while intermediate rates typically occur in Southeast Asia, and South American populations of lowest endemicity tend to have a more ‘clonal’ structure with occasional recombination [21]–[24]. On top of this inter-continental trend, there is a variety of population structures in the diverse endemic foci of Southeast Asia [21], [25] and South America [21], [24], and evidence that continental Africa may contain some populations with a relatively low effective recombination rate [22], [26].

Because of this spectrum, despite limited knowledge of variation in LD throughout the *P. falciparum* genome [27], it could be useful to first screen for allelic associations in a population of low to moderate endemicity (in which LD will tend to be more extensive), and subsequently attempt confirmation and finer mapping in a more highly endemic population with less LD. Thailand is a country mostly free of malaria, but it still contains some areas of moderate endemicity from which large numbers of severe as well as mild malaria cases have been admitted into well managed hospital facilities [28].

Here, to investigate the potential for allelic association studies in Thailand, an analysis of linkage disequilibrium was first undertaken with a sample of 100 *P. falciparum* clinical isolates, using one set of microsatellite loci widely distributed in the genome, and another set clustered within a ∼140 kb region of one chromosome. Results show that LD is weak and rarely significant above distances of ∼5 kb, so allelic association requires genotype information from within genes that contain causally relevant variants. The second phase of the study then involved a disease association analysis comparing a sample of 113 severe malaria cases and 245 mild malaria controls for polymorphisms in six candidate merozoite stage genes (three *eba* and three *Rh* genes) previously shown to be important in determining erythrocyte invasion phenotypes *in vitro* [29] and thus potentially related to parasite growth and virulence in *vivo*.

## Methods

### Ethics Statement

The proposal was approved by the Ethics Review Committee of the Faculty of Tropical Medicine, Mahidol University. Participants gave written informed consent to provide a blood sample for studies including analysis of parasite DNA.

### P. falciparum DNA samples from malaria cases

Patients with *P. falciparum* malaria presented at the Hospital for Tropical Diseases, Mahidol University, Bangkok. For the initial study of linkage disequilibrium, blood samples were selected from 100 patients who presented in 1999 with uncomplicated malaria, having acquired infection in the Thai-Myanmar border region. For the severe malaria case-control study, 113 patients with severe malaria as defined by criteria from WHO that were admitted to the Hospital for Tropical Diseases, and 245 mild malaria patients with uncomplicated non-severe malaria (termed ‘mild malaria’) were recruited in 2002 and 2003. Mild malaria patients were recruited either from the ward or from the outpatient department of the Hospital for Tropical Diseases, matching to a severe case by residential location. Exclusion criteria included pregnancy, mixed malaria species infection, HIV infection, and reported prior treatment with any antimalarial drug. After consent, 5 ml of venous blood was drawn from each participant, of which 1 ml was collected in an EDTA tube and stored at −20°C prior to extraction of DNA using QIAamp DNA Blood Mini Kits (Qiagen).

### Microsatellite genotyping

**Figure 1:**
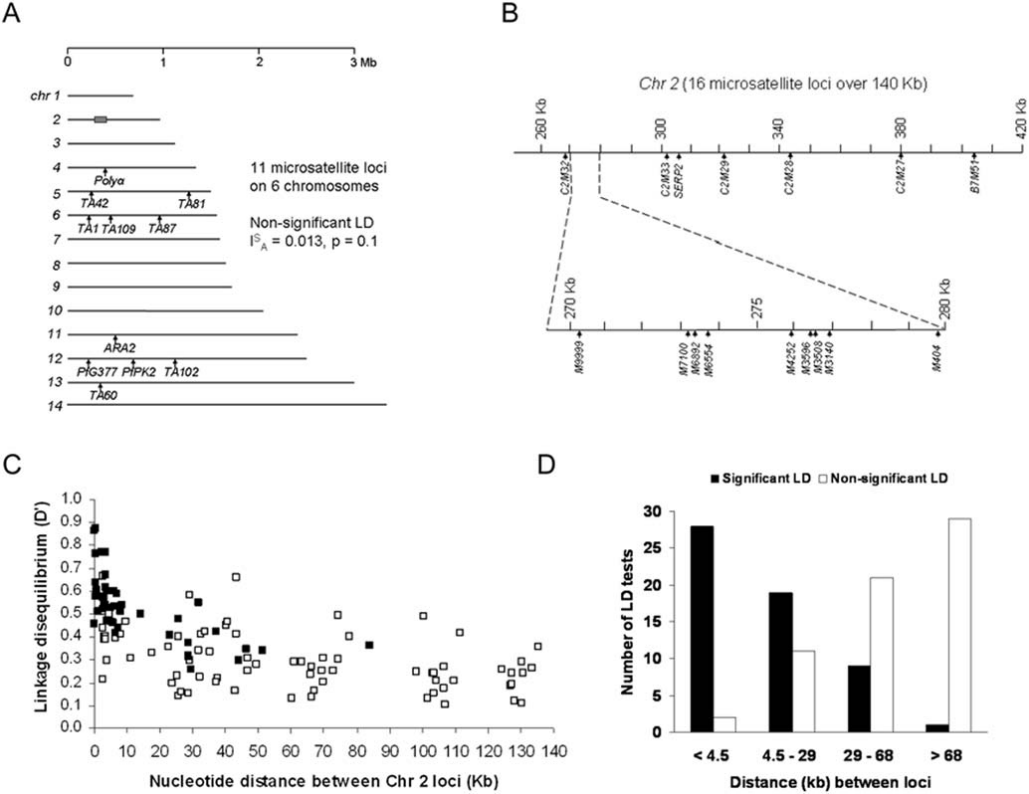
A. Positions of 11 microsatellite loci on 6 of the 14 *P. falciparum* chromosomes (*chr 4–6* and *11–13*) studied in a multi-locus test of LD that assessed the standardised index of associations among all alleles (*I* ^S^_A_). The shaded box on *chr 2* shows the position of a region that was subsequently studied. B. Positions of 16 microsatellite loci within a 140 kb region of *chr 2*: The top line shows positions of 7 microsatellites that were previously identified and broadly cover the region; the bottom line shows positions of 9 microsatellites that were newly identified within a ∼10 kb sub-region between positions 270 to 280 kb (all positions are numbered according to the *chr 2* reference sequence of isolate 3D7). C. Relationship between strength of LD (D′ index, mean value for alleles of frequency >0.1 in each pair of loci) and physical distance between pairs of microsatellite loci in a 135 kb region of *chr 2*. Each filled square indicates a pair of loci for which one or more allelic association was significant, while open squares show pairs of loci that were non-significantly associated. D. Significance of LD as proportions of pairs of loci with one or more significant association between tested alleles (Fisher's exact test on alleles of frequency >0.1) for pairs of loci separated by different map distances (N = 30 for each of the quartiles).

Eleven microsatellite loci widely separated in the P. *falciparum* genome (Figure 1A) were typed: Polyα (chr 4), TA42 (chr 5), TA81 (chr 5), TA1 (chr 6), TA87 (chr 6), TA109 (chr 6), ARA2 (chr 11), Pfg377 (chr 12), PfPK2 (chr 12), TA102 (chr 12), TA60 (chr 13), using methods previously described [30]. Of these, the few loci on the same chromosomes are well separated (the closest being TA1 and TA109 which are >100 kb apart on chr 6, ∼10 cM in an experimental genetic cross) [16]. Next, sixteen microsatellite loci that map within a 140 Kb region of chr 2 (Figure 1B) were typed: seven (C2M32, C2M33, SERP2, CM29, CM28, CM27 and B7M51) were from a genome-wide microsatellite map (http://www.ncbi.nlm.nih.gov/Malaria/Mapsmarkers/PfGMap/pfgmap_2.html) [16], and nine (M404, M3140, M3508, M3596, M4252, M6554, M6892, M7100 and M9999) were identified by alignment of ∼10 Kb sequence of the parasite line B8 (Accession number AF033037) with the corresponding sequence from 3D7 chromosome 2 (AE001362) (this region was chosen as, prior to recent genome-wide shotgun sequencing efforts, it contained the longest contiguous sequence from different isolates to allow discovery of new polymorphic markers). PCR primer sequences and dye labels are shown in Supplementary Table S1. Products were run on an ABI PRISM 377 DNA sequencer with size standards (ROX-350), and Genescan 3.1 and Genotyper 2.0 software (Applied Biosystems, UK) were used for allele scoring.

### Analysis of multi-locus LD

For each locus in each isolate, a single allele (the only allele detected, or the predominant allele in an isolate containing more than one genotype) was counted. For the analysis of multi-locus LD among the 11 widely separated microsatellites, the standard index of association (*I^S^_A_*), was calculated and tested for departure from random allelic association using LIAN 3.0 software [31]. Multiple-clone isolates containing more than one allele at any locus were not included in this analysis. For the 16 *chr 2* microsatellites, the *D′* pairwise index of linkage disequilibrium (LD) was calculated using alleles that had a frequency of >0.1, and mean values were derived for each pair of loci with multiple alleles. Data on all isolates were incorporated, but individual pairwise genotype data points were excluded if both loci had mixed alleles in a given isolate. The relationship between LD and distance between nucleotide sites was plotted, and the proportion of pairs of loci that had statistically significant LD was compared for increasing distance categories by Fisher's exact test.

### Genotyping polymorphisms in the eba and Rh genes

**Figure 2:**
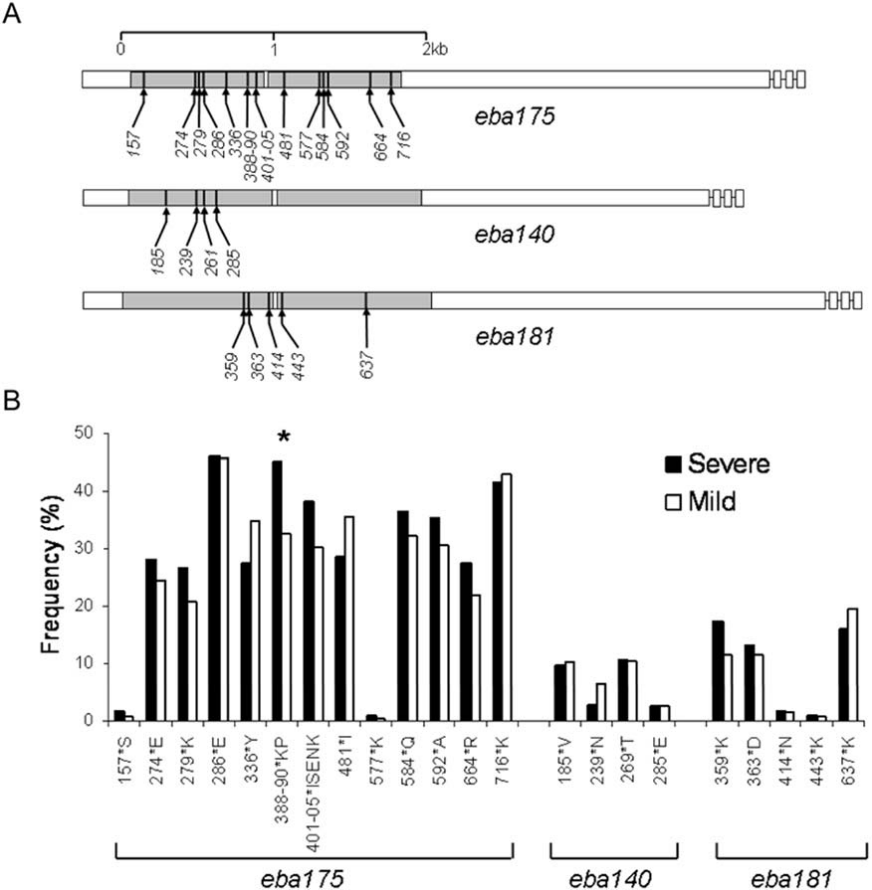
A. Positions of polymorphic sites genotyped in Region II of each of the three protein coding *eba* genes: *eba175* (chromosome, chr 7), *eba140* (chr 13) and *eba181* (chr 1). Most polymorphisms were individual coding SNPs labelled here as the number of the codon in the sequence of the corresponding protein, although some covered two SNPs or a cluster of SNPs and an indel (sequences of 18-mer allele-specific oligonucleotide probes used are given in Supplementary Table 2). B. Frequencies of the second most common allele at each polymorphic site in severe malaria cases and mild malaria controls. Significant difference between the groups is shown with an asterisk (*).

**Figure 3:**
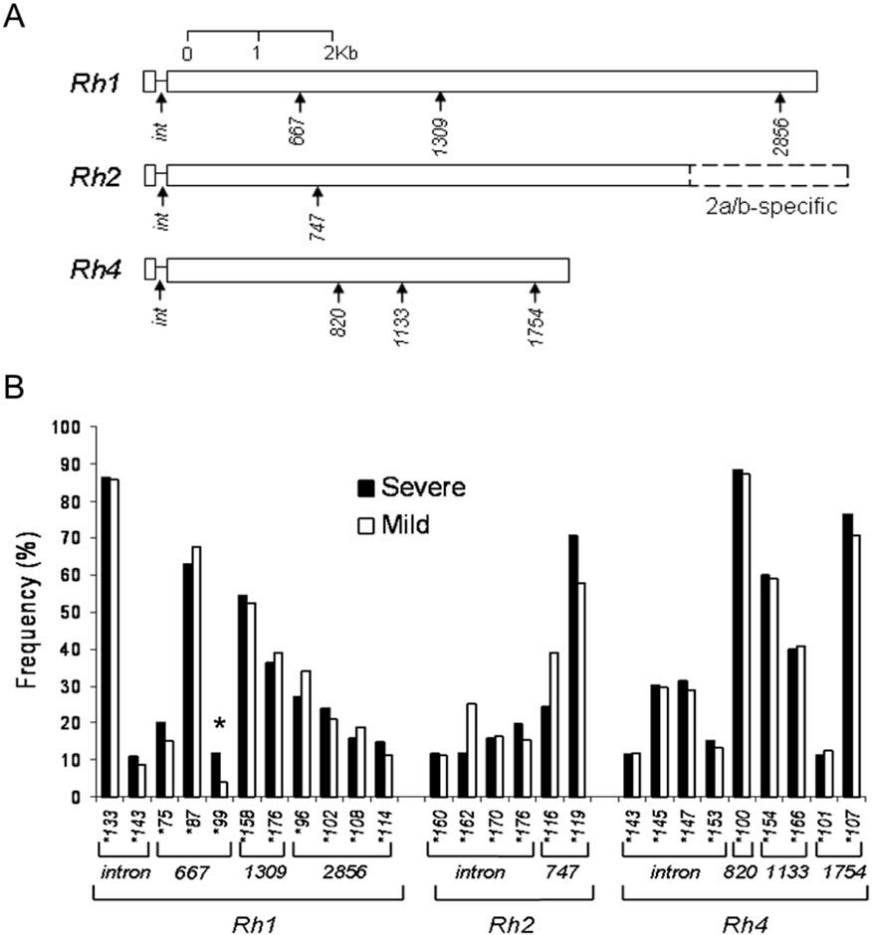
A. Positions of microsatellites genotyped within the protein coding *Rh* genes: *Rh1* (chromosome, chr 4) , *Rh2(a/b)* (chr 13), and *Rh4* (chr 4). The *Rh2* locus contains 2 adjacent genes (*a* and *b*) with most sequence in common and the two polymorphisms typed here are in the region with haplotypes shared between the genes by frequent gene conversion (the region specific to locus a or b was not investigated). B. Frequencies of the alleles in severe malaria cases (rare alleles with a frequency <10% in this group are not shown) and mild malaria controls. Significant difference between the groups is shown with an asterisk (*).

Polymorphic sites in *eba175* (*chr 7*), *eba140* (*chr 13*), and *eba181* (*chr 1*) were studied (Figure 2A), including 11 loci in *eba175* (9 separate SNPs, a pair of closely situated SNPs, and a run of SNPs next to an indel), 4 SNP loci in *eba140* gene, and 3 SNP loci in *eba181* gene, by allele sequence-specific oligonucleotide probing of PCR products (PCR-SSOP) following methods described for other loci [22] (specific primer and probe sequences and conditions are given in Supplementary Table S2). A set of polymorphic simple sequence repeat loci was chosen from allele sequence alignments of the *Rh1* gene (*chr* 4), the identical part of the adjacent *Rh2a* and *Rh2b* genes (*chr* 13), and the *Rh4* gene (*chr* 4) (Figure 3A). Oligonucleotide primers and fluorescent labels used are listed in Supplementary Table S3. Size standards (500 LIZ®, Applied Biosystems, UK), were run together with PCR products on an ABI PRISM™ 3730 Genetic Analyzer, and the GeneMapper™ 3.0 program (Applied Biosystems, UK) was used for automated measurement of allele length and peak height.

## Results

### Multi-locus LD analysis of microsatellites on different chromosomes

Complete genotype data for 11 widely separated microsatellite loci (Figure 1A) were obtained for 98 of 100 Thai *P. falciparum* isolates tested, with numbers of alleles per locus ranging from 4 in *Pfg377* up to 17 in *Polyα* (allele frequencies are shown in Supplementary Figure S1). Fifty of these isolates had single alleles at each of the loci indicating unmixed haploid genotype infections, and these were suitable for analysis of multilocus linkage disequilibrium, as they exclude possibility of scoring a false haplotype from the presence of mixed genotypes. The standardised index of association (*I^S^_A_*) (a multi-locus test of LD among all the 11 loci) showed a low value, *I^S^_A_* = 0.013, not significantly different from the randomly expected value of zero (*P* = 0.1), indicating very little LD amongst unlinked loci.

#### LD among microsatellite loci in a 140 kb region of chr 2

The next analysis of LD in the same set of 100 Thai isolates focused on a representative non-telomeric part of the genome for which a dense set of microsatellite markers could be identified (as described in the Methods section). For 16 microsatellite loci within a 140 kb region of *chr* 2 (Figure 1B), numbers of alleles per locus ranged from 2 (*M3596*) to 20 (*M6554*) (allele frequencies are shown in Supplementary Figure S2). For each of the 120 pairs among the 16 loci, the *D́* linkage disequilibrium index was plotted (using the values for each pair of alleles that had frequencies of >0.1 and taking the mean wherever there were multiple alleles at either or both loci). As expected, the strength and significance of linkage disequilibrium was inversely related to physical distance (Figure 1C). The 120 pairs of loci were ordered into four equal sized categories (n = 30) representing the quartiles of pairwise data (<4.5 kb, 4.5–29 kb, 29–68 kb, and >68 kb), revealing highly significant declines in significant LD throughout the distance range (Figure 1D). There was a much higher proportion of significant LD in the smallest distance category of <4.5 kb (93%) than over the next distance category of 4.5–29 kb (63%, *P* = 0.0051). Moreover, the proportion of significant tests in the 4.5–29 kb category was much higher than in the next category of 29–68 kb (30%, *P* = 0.0097), and the latter proportion was higher than in the >68 kb category (3%, *P* = 0.0061).

### Allelic association of candidate genes in a severe malaria case-control study

As the average gene density in the *P. falciparum* genome is one every ∼4.5 kb [32], LD extending beyond individual genes will generally be very weak, so genome-wide approaches will need adequate markers within every gene. The next stage of the present study focused on polymorphisms within a set of candidate virulence genes. Six genes encoding merozoite ligands that use alternative receptors for erythrocyte invasion were investigated in a case-control study of severe malaria (n = 113 cases) compared with mild malaria (n = 245 controls). For three *eba* genes (*eba175*, *eba140* and *eba181*), most of the previously identified polymorphisms encoding amino acid changes within the erythrocyte binding domain (Region II) of each protein were genotyped (Figure 2A). For three *Rh* genes (*Rh1*, *Rh2a/b*, and *Rh4*) microsatellite loci within the intron and coding sequence of each gene were identified and genotyped (Figure 3A). A single allele per locus per isolate (the only detectable allele, or most abundant allele in isolates which contain more than one genotype) was counted for the analysis comparing frequencies between severe and mild malaria groups.

The case and control groups were well matched for most variables, but there were frequency differences between the groups in gender, year of recruitment, and history of having malaria previously (Table 1), so these variables were incorporated in a logistic regression analysis with any polymorphisms that emerged as different between the groups in a univariate analysis (Tables 2 and 3). After this adjustment, there were significant differences between severe and mild malaria groups for one polymorphism in *eba175* (Figure 2B and Table 2) and one in *Rh1* (Figure 3B and Table 3). The *eba175* allele KP at codons 388 and 390 was significantly more common in severe than mild malaria (45% versus 32%), with an odds ratio (OR) of 1.93 (95% CI, 1.18–3.17) compared to the common allele (*P* = 0.009). The 99bp allele in the polymorphic repeat centred on codon 667 of *Rh1* was more common in severe than mild malaria (12% versus 4%), with an OR of 3.14 (95% CI 1.20–8.19) compared to the most common allele (*P* = 0.02). In the 5′ region of *Rh2a/b* there were alleles that had borderline significant univariate associations with severe malaria (in the intron and the microsatellite centred on codon 747), but these did not remain significant after adjustment for potential confounding by the logistic regression analysis (Table 3). The allele frequencies for all other polymorphisms in these genes and in *eba140*, *eba181*, *Rh4* were similar between the groups.

**Table 1:** Demographic profile of malaria patients recruited into the case-control study

	Uncomplicated malaria (*n = *245)	Severe malaria (*n = *113)	Univariate odds ratio OR (95% CI)	P value
**Gender**				
Male	187 (76.3)	68 (61.7)	1	0.002 *
Female	58 (23.7)	45 (39.8)	2.13 (1.28–3.53)	
**Age group**				
5–14	7 ( 2.9)	2 ( 1.77)	0.65 (0.13–3.24)	0.96
15–24	123 (50.2)	54 (47.79)	1	
25–34	70 (28.6)	36 (31.86)	1.17 (0.70–1.96)	
35–44	31 (12.7)	14 (12.39)	1.03 (0.51–2.09)	
>45	14 (5.7)	7 ( 6.19)	1.14 (0.44–2.98)	
**Year**				
2002	144 (58.8)	50 (44.25)	1	0.01 *
2003	101 (41.2)	63 (55.75)	1.80 (1.15–2.82)	
**Region infection acquired** §	(*n = *241)	(*n = *113)		
North-West	121 (50.2)	48 (42.48)	1	0.12
West	115 (47.7)	58 (51.33)	1.27 (0.80–2.01)	
Eastern	2 (0.8)	4 ( 3.54)	5.04 (0.89–28.4)	
Central	3 (1.2)	3 ( 2.65)	2.52 (0.49–12.9)	
**Previous malaria**	(*n = *245)	(*n = *111)		
No	134 (54.7)	93 (82.0)	1	<0.001 *
Yes	111 (45.3)	20 (18.0)	0.27 (0.15–0.46)	
**Ethnic group**	(*n = *244)	(*n = *112)		
Maun	81 (33.2)	46 (41.1)	1	0.28
Burmese	56 (23.0)	16 (14.3)	0.50 (0.26–0.98)	
Thai	55 (22.5)	24 (21.4)	0.77 (0.50–1.63)	
Karen	51 (20.9)	26 (23.2)	0.90 (0.50–1.63)	
Thaiyai	1 ( 0.4)	0 (0)	0	

§ Provinces in north-western part of Thailand (Tak, Mae Hongson, Chiengrai, Chiengmai, and Myanmar border), western part (Prajuabkirikun, Ratchaburi, Kanchanaburi), eastern areas (Prajeanburi, Chanthaburi, Trad and Cambodia border), and central part of Thailand (Chaiyapoom, Kumpangpet, Lumpang, Nakornnayok, and Phitsanulok).

* Variables that were significantly different between the groups (gender, year, and history of previous malaria) were adjusted for in the case-control analysis of parasite polymorphisms.

**Table 2:** Alleles of *eba-140*, *eba-181, eba-175* in the severe malaria case-control study

*Locus, Allele*	Severe Malaria patients (%)	Mild Malaria patients (%)	Crude Odds Ratio (95% CI); significant *P value*	Adjusted Odds Ratio (95% CI); significant *P value*
***eba140***				
*codon 185*	*n = *113	*n* = 243		
*I	102 (90.3)	218 (89.7)	1	
*V	11 (9.7)	25 (10.3)	0.94 (0.40–2.07)	
*codon 239*	*n* = 113	*n* = 243		
*S	110 (97.4)	227 (93.4)	1	
*N	3 (2.7)	16 (6.6)	0.39 (0.09–1.45)	
*codon 261*	*n* = 113	*n* = 241		
*K	92 (81.4)	202 (83.8)	1	
*R	9 (8.0)	14 (5.8)	1.41 (0.59–3.38)	
*T	12 (10.6)	25 (10.4)	1.05 (0.51–2.19)	
*codon 285*	*n* = 113	*n* = 242		
*K	110 (97.4)	236 (97.5)	1	
*E	3 (2.7)	6 (2.5)	1.07 (0.17–5.13)	
***eba181***				
*codon 359*	*n* = 98	*n* = 209		
*R	81 (82.7)	185 (88.5)	1	
*K	17 (17.4)	24 (11.5)	1.62 (0.77–3.33)	
*codon 363*	*n* = 113	*n* = 244		
*V	98 (86.7)	216 (88.5)	1	
*D	15 (13.3)	28 (11.5)	1.18 (0.56–2.40)	
*codon 637*	*n* = 87	*n* = 179		
*N	73 (83.9)	144 (80.5)	1	
*K	14 (16.1)	35 (19.5)	0.79 (0.37–1.62)	
***eba175***				
*codon 274*	*n* = 103	*n* = 235		
*K	74 (71.8)	177 (75.3)	1	
*E	29 (28.2)	58 (24.7)	1.20 ( 0.68–2.07)	
*codon 279*	*n* = 113	*n* = 245		
*E	83 (73.5)	194 (79.2)	1	
*K	30 (26.5)	51 (20.8)	1.37 (0.79–2.38);	
*codon 286*	*n* = 113	*n* = 245		
*K	61 (54.0)	133 (54.3)	1	
*E	52 (46.0)	112 (45.7)	1.01 (0.63–1.62)	
*codon 336*	*n* = 113	*n* = 245		
*D	82 (72.6)	160 (65.3)	1	
*Y	31 (27.4)	85 (34.7)	0.71 (0.42–1.19)	
*codon 388-90*	*n* = 113	*n* = 245		
*NS	57 (50.4)	160 (65.3)	1	1
*KP	51 (45.1)	80 (32.7)	1.79 (1.13–2.84); P = 0.01**	1.93 (1.18–3.17); P = 0.009**
*KS	5 (4.4)	5 (2.0)	2.81 (0.78–10.05)	1.94 (0.53–7.07)
*codon 401-5*	*n* = 113	*n* = 245		
*ISKNK	39 (34.5)	89 (36.3)	1	
*–KKM	31 (27.4)	82 (33.5)	0.86 (0.49–1.51)	
*ISENK	43 (38.1)	74 (30.2)	1.33 (0.78–2.26)	
*codon 481*	*n* = 112	*n* = 216		
*K	80 (71.4)	139 (64.4)	1	
*I	32 (28.6)	77 (35.6)	0.72 (0.42–1.22)	
*codon 584*	*n* = 113	*n* = 239		
*K	34 (30.1)	87 (36.4)	1	
*E	38 (33.6)	75 (31.4)	1.30 (0.74–2.26)	
*Q	41 (36.3)	77 (32.2)	1.36 (0.79–2.36)	
*codon 592*	*n* = 113	*n* = 244		
*E	73 (64.6)	169 (69.3)	1	
*A	40 (35.4)	75 (30.7)	1.23 (0.75–2.03)	
*codon 664*	*n* = 113	*n* = 242		
*S	82 (72.6)	189 (78.1)	1	
*R	31 (27.4)	53 (21.9)	1.35 (0.78–2.32)	
*codon 716*	*n* = 113	*n* = 240		
*E	66 (58.4)	137 (57.1)	1	
*K	47 (41.6)	103 (42.9)	0.95 (0.59–1.53)	

The common allele at each locus is the reference allele (Odds Ratio = 1). Adjusted odds ratios result from a multivariate analysis adjusting for gender, previous history of ever having malaria, and the year of recruitment.

**Table 3:** Alleles of *Rh1*, *Rh2(a,b)*, and *Rh4* in the severe malaria case-control study

*Locus, Allele*	Severe Malaria patients (%)	Mild Malaria patients (%)	Crude Odds Ratio (95% CI); significant *P value*	Adjusted Odds Ratio (95% CI); significant *P value*
***Rh1***				
*intron repeat*	*n* = 80	*n* = 193		
*133	70 (87.5)	166 (86.0)	1	
*123	1 (1.3)	10 (5.2)	0.24 (0.03–1.89)	
*143	9 (11.3)	17 (8.8)	1.26 (0.53–2.95)	
*codon 667 repeat*	*n* = 100	*n* = 228		
*87	63 (63.0)	155 (68.0)	1	1
*75	20 (20.0)	35 (15.4)	1.41 (0.75–2.62)	1.40 (0.72–2.71)
*93	5 (5.0)	20 (8.8)	0.62 (0.22–1.71)	0.78 (0.27–2.28)
*99	12 (12.0)	9 (3.9)	3.28 (1.32–8.17); P = 0.01**	3.14 (1.20–8.19); P = 0.02**
*105	0 (0)	9 (3.9)	0	
*codon 1309 repeat*	*n* = 99	*n* = 229		
*158	54 (54.6)	120 (52.4)	1	
*140	9 (9.1)	20 (8.7)	1.00 (0.43–2.34)	
*176	36 (36.4)	89 (38.9)	0.90 (0.54–1.49)	
*codon 2856 repeat*	*n* = 100	*n* = 227		
*96	27 (27.0)	78 (34.4)	1	
*90	8 (8.0)	11 (4.8)	2.10 (0.76–5.77)	
*102	24 (24.0)	48 (21.1)	1.44 (0.75–2.79)	
*108	16 (16.0)	43 (18.9)	1.07 (0.52–2.21)	
*114	15 (15.0)	26 (11.4)	1.67 (0.77–3.61)	
*120	8 (8.0)	14 (6.2)	1.65 (0.62–4.37)	
*138	2 (2.0)	7 (3.1)	0.83 (0.16–4.22)	
***Rh2 (a,b)***				
*Intron repeat*	*n* = 66	*n* = 169		
*162	9 (13.6)	46 (27.2)	1	1
*160	9 (13.6)	21 (12.4)	2.19 (0.76–6.31)	1.85 (0.60–5.67)
*164	5 (7.8)	10 (5.9)	2.56 (0.70–9.28)	2.71 (0.68–10.87)
*168	5 (7.8)	9 (5.3)	2.84 (0.77–10.48)	2.48 (0.63–9.82)
*170	12 (18.2)	30 (17.8)	2.04 (0.77–5.44)	1.77 (0.63–4.94)
*172	5 (7.8)	8 (4.7)	3.19 (0.85–12.03)	2.80 (0.68–11.58)
*174	6 (9.1)	17 (10.1)	1.80 (0.56–5.83)	1.36 (0.38–4.94)
*176	15 (22.7)	28 (16.6)	2.74 (1.06–7.08); P = 0.04**	2.56 (0.94–7.01); P = 0.07
*codon 747 repeat*	*n* = 81	*n* = 195		
*119	58 (71.6)	114 (58.5)	1	1
*110	3 (3.7)	4 (2.1)	1.47 (0.32–6.81)	1.34 (0.27–6.75)
*116	20 (24.7)	77 (39.5)	0.51 (0.28–0.92); P = 0.02**	0.56 (0.30–1.04); P = 0.07
***Rh4***				
*intron repeat*	*n* = 87	*n* = 201		
*145	26 (29.9)	60 (29.9)	1	
*143	10 (11.5)	24 (11.9)	0.96 (0.40–2.29)	
*147	27 (31.0)	59 (29.4)	1.06 (0.55–2.02)	
*149	1 (1.1)	10 (5.0)	0.23 (0.03–1.90)	
*153	13 (14.9)	27 (13.4)	1.11 (0.50–2.49)	
*155	8 (9.2)	21 (10.4)	0.88 (0.35–2.24)	
*codon 820 repeat*	*n* = 95	*n* = 217		
*100	84 (88.4)	191 (88.0)	1	
*91	4 (4.2)	9 (4.1)	1.01 (0.30–3.37)	
*97	1 (1.1)	6 (2.8)	0.38 (0.05–3.20)	
*103	6 (6.3)	11 (5.1)	1.24 (0.44–3.46)	
*codon 1133 repeat*	*n* = 95	*n* = 195		
*154	57 (60.0)	115 (59.0)	1	
*166	38 (40.0)	80 (41.0)	0.96 (0.56–1.63)	
*codon 1754 repeat*	*n* = 94	*n* = 223		
*107	73 (77.7)	160 (71.7)	1	
*101	11 (11.7)	28 (12.6)	0.86 (0.41–1.82)	
*113	9 (9.6)	31 (13.9)	0.64 (0.29–1.41)	
*119	1 (1.1)	4 (1.8)	0.55 (0.06–4.99)	

The common allele at each locus is the reference allele (Odds Ratio = 1). Adjusted odds ratios result from a multivariate analysis adjusting for gender, previous history of ever having malaria, and the year of recruitment.

The two significant associations were examined further. In *eba175*, codons 388/390 KP are always present with particular alleles at flanking polymorphisms, consistent with a recent sequence analysis in which an indel at codons 401–402 (IS/–) was in complete LD with 336 D, 388/390 KP, and 403-5 ENK in Thailand [33], but not in a Kenyan [33] or Nigerian [34] population. Here, haplotypes were derived from genotypes of single clone infection isolates (excluding any isolates with mixed alleles detected). After adjusting for potential confounding, the most common codon 336–405 haplotype DKPISENK was over-represented among severe malaria cases (40%, 41/102) compared with mild malaria controls (30%, 69/231), with an OR of 1.68 (95% CI, 1.01–2.81; P = 0.047 compared with other haplotypes combined). Frequencies of two other common haplotypes DNS–KKM and YNSISKNK were not different from those in the mild malaria group. In *Rh1*, the polymorphism centred on codon 667 is not in strong LD with the flanking polymorphisms typed that are respectively ∼2 kb upstream (intron repeat) and ∼2 kb downstream (repeat centred on codon 1309).

## Discussion

An earlier survey of unlinked microsatellites in parasites sampled over a single month in a small area on the Thai-Burma border (Shoklo) showed a higher index of association (multilocus LD) than seen here, but this difference disappeared when only unique haplotypes were analysed in that study, indicating a locally ‘epidemic’ population structure [21]. The absence of such a population structure in the present study is more typical of a broad population sample that would be suitable for association studies. The modest LD in this study, rarely extending beyond 5 kb, confirms that genome-wide allelic association studies will require a very dense set of markers covering all genes. A current effort has already discovered >50,000 SNPs [17]–[19] (data available at www.plasmodb.org), and further discovery is ongoing with an aim to eventually design a more dense genotyping array [35]. It is possible that a genome-wide array of ∼50,000 SNPs would capture sufficient LD for population based association studies to be attempted in Thailand. If half of the globally discovered SNPs were polymorphic in Thailand, so that ∼25,000 SNPs were typed with such an array, the SNP marker density would average ∼1 per kb, at which distance pairwise LD indices in the Thai *P. falciparum* population are often moderately strong (D′>0.5) [23], [33], [36].

Most African *P. falciparum* populations will have less LD than the Thai parasite population [21]–[23], [27], so an initial screen for allelic associations in Africa with the same SNP marker density would be less likely to score a ‘hit’, although it might pick up alleles that have recently been under strong positive selection and have led to a selective sweep of associated haplotypes, of which drug resistance alleles are extreme examples [37]–[39]. Commitment to discovering and developing higher density SNP genotyping arrays is therefore necessary for association studies in Africa, and would also allow the benefit of finer mapping of associated polymorphisms. In practice, it would be difficult to conduct a very large case-control study of severe malaria outside of Africa due to limited numbers of severe cases in any one population, although a multi-centre study in Southeast Asia might be feasible [28]. Even for studies within Africa, a large consortial approach would help to achieve a desirable sample size of more than 1000 *P. falciparum* isolates from severe cases compared with at least an equivalent number of mild controls, and ideally also asymptomatic infected controls. Ultimately it would be beneficial to also compare results across populations that have somewhat different haplotype structure in the parasite populations, as is now being applied to human genetic studies of common diseases [40], [41].

The polymorphisms in six candidate parasite virulence genes tested here in a modestly sized severe malaria case-control study yielded two associations at a P<0.05 level of significance, unadjusted for multiple hypothesis testing. These could have arisen by chance given the number of loci tested, and should only be considered as preliminary ‘hits’ for further examination. The significant disease association in *eba175* tags a short haplotype in the middle of the binding region II incorporating a 2-codon indel and flanking amino acid polymorphisms in the c-terminal part of the F1 sub-domain, immediately before the hinge region that precedes the F2 sub-domain [42]. The disease association in *Rh1* at the microsatellite centred on codon 667 may tag a region encoding a binding domain or a target of immunity (neither of the flanking microsatellites ∼2 kb on either side showed an association). This part of the Rh1 molecule contains a number of amino acid polymorphisms [43], and most significantly, a recent study has identified it as an important erythrocyte binding domain against which antibodies can block invasion [44].

Polymorphisms in *eba140* and *eba181* previously reported to affect specificity of erythrocyte binding in a transfected COS cell assay [45], [46] were tested but not associated with severe malaria here. These polymorphisms were not shown to be associated with erythrocyte invasion phenotypes among isolates in Brazil [47]. The polymorphisms in the 5′-part of the *Rh2* gene sequence that is shared between paralogues *Rh2a* and *Rh2b* showed marginal associations with disease that were not significant after adjusting for potential confounders between the groups. Polymorphisms that are specific for *Rh2a* and *Rh2b* towards the 3′-end of each gene may be associated with differences in erythrocyte invasion phenotype, as suggested for *Rh2b* in a recent study in Senegal [48] and in Brazil [49], and should be tested in future disease association studies. None of the *Rh4* polymorphisms tested here showed disease associations, although there are differences between parasite lines in their ability to switch to *Rh4*-dependent invasion that involves a neuraminidase-resistant erythrocyte receptor [50], [51]. An adjacent paralogous gene *Rh5* has very recently been shown to encode a smaller protein with amino acid polymorphisms that affect erythrocyte receptor binding [52], and is a candidate for future studies. It is also possible that non-coding polymorphisms influence transcription of these genes in cis- or trans-acting regulatory mechanisms, and these would be among the potentially important loci to be discovered by a genome-wide approach.

## Supporting Information

Table S1 (0.10 MB DOC)

Table S2 (0.15 MB DOC)

Table S3 (0.14 MB DOC)

Figure S1 Allele frequencies at 11 widely separated microsatellite loci in 100 Plasmodium falciparum isolates in Thailand
(0.06 MB PPT)

Figure S2 Allele frequencies at 16 microsatellite loci in a 140 kb region of Chromosome 2 in a sample of 100 Plasmodium falciparum isolates in Thailand (asterisks show alleles used in LD analyses)
(0.08 MB PPT)

## References

[1] Rowe AK, Rowe SY, Snow RW, Korenromp EL, Schellenberg JR (2006). The burden of malaria mortality among African children in the year 2000.. Int J Epidemiol.

[2] Vannaphan S, Saengnetswang T, Suwanakut P, Kllangbuakong A, Klinnak W (2005). The epidemiology of patients with severe malaria who died at the Hospital for Tropical Diseases, 1991–2004.. Southeast Asian J Trop Med Public Health.

[3] de Roode JC, Pansini R, Cheesman SJ, Helinski ME, Huijben S (2005). Virulence and competitive ability in genetically diverse malaria infections.. Proc Natl Acad Sci U S A.

[4] Otsuki H, Kaneko O, Thongkukiatkul A, Tachibana M, Iriko H (2009). Single amino acid substitution in *Plasmodium yoelii* erythrocyte ligand determines its localization and controls parasite virulence.. Proc Natl Acad Sci USA, epub ahead of print.

[5] Pattaradilokrat S, Culleton RL, Cheesman SJ, Carter R (2009). Gene encoding erythrocyte binding ligand linked to blood stage multiplication rate phenotype in *Plasmodium yoelii yoelii*.. Proc Natl Acad Sci USA, epub ahead of print.

[6] Mackinnon MJ, Read AF (2004). Virulence in malaria: an evolutionary viewpoint.. Philos Trans R Soc Lond B Biol Sci.

[7] Ariey F, Hommel D, Le Scanf C, Duchemin JB, Peneau C (2001). Association of severe malaria with a specific *Plasmodium falciparum* genotype in French Guiana.. J Infect Dis.

[8] Kun JF, Schmidt-Ott RJ, Lehman LG, Lell B, Luckner D (1998). Merozoite surface antigen 1 and 2 genotypes and rosetting of *Plasmodium falciparum* in severe and mild malaria in Lambarene, Gabon.. Trans R Soc Trop Med Hyg.

[9] Engelbrecht F, Felger I, Genton B, Alpers M, Beck H.-P (1995). *Plasmodium falciparum*: malaria morbidity is associated with specific merozoite surface antigen 2 genotypes.. Exp Parasitol.

[10] Robert F, Ntoumi F, Angel G, Candito D, Rogier C (1996). Extensive genetic diversity of Plasmodium falciparum isolates collected from patients with severe malaria in Dakar, Senegal.. Trans R Soc Trop Med Hyg.

[11] Cramer JP, Mockenhaupt FP, Mohl I, Dittrich S, Dietz E (2004). Allelic dimorphism of the erythrocyte binding antigen-175 (eba-175) gene of *Plasmodium falciparum* and severe malaria: Significant association of the C-segment with fatal outcome in Ghanaian children.. Malar J.

[12] Toure FS, Bisseye C, Mavoungou E (2006). Imbalanced distribution of *Plasmodium falciparum* EBA-175 genotypes related to clinical status in children from Bakoumba, Gabon.. Clin Med Res.

[13] Amodu OK, Oyedeji SI, Ntoumi F, Orimadegun AE, Gbadegesin RA (2008). Complexity of the msp2 locus and the severity of childhood malaria, in south-western Nigeria.. Ann Trop Med Parasitol.

[14] Normark J, Nilsson D, Ribacke U, Winter G, Moll K (2007). PfEMP1-DBL1alpha amino acid motifs in severe disease states of *Plasmodium falciparum* malaria.. Proc Natl Acad Sci USA.

[15] Bull PC, Buckee CO, Kyes S, Kortok MM, Thathy V (2008). *Plasmodium falciparum* antigenic variation. Mapping mosaic *var* gene sequences onto a network of shared, highly polymorphic sequence blocks.. Mol Microbiol.

[16] Su X.-Z, Ferdig MT, Huang Y, Huynh CQ, Liu A (1999). A genetic map and recombination parameters of the human malaria parasite *P. falciparum*.. Science.

[17] Volkman SK, Sabeti PC, DeCaprio D, Neafsey DE, Schaffner SF (2007). A genome-wide map of diversity in *Plasmodium falciparum*.. Nat Genet.

[18] Jeffares DC, Pain A, Berry A, Cox AV, Stalker J (2007). Genome variation and evolution of the malaria parasite *Plasmodium falciparum*.. Nat Genet.

[19] Mu J, Awadalla P, Duan J, McGee KM, Keebler J (2007). Genome-wide variation and identification of vaccine targets in the *Plasmodium falciparum* genome.. Nat Genet.

[20] Su X, Hayton K, Wellems TE (2007). Genetic linkage and association analyses for trait mapping in *Plasmodium falciparum*.. Nat Rev Genet.

[21] Anderson TJC, Haubold B, Williams JT, Estrada-Franco JG, Richardson L (2000). Microsatellites reveal a spectrum of population structures in the malaria parasite *Plasmodium falciparum*.. Mol Biol Evol.

[22] Conway DJ, Roper C, Oduola AMJ, Arnot DE, Kremsner PG (1999). High recombination rate in natural populations of *Plasmodium falciparum*.. Proc Natl Acad Sci USA.

[23] Polley SD, Chokejindachai W, Conway DJ (2003). Allele frequency based analyses robustly identify sites under balancing selection in a malaria vaccine candidate antigen.. Genetics.

[24] Machado RL, Povoa MM, Calvosa VSP, Ferreira MU, Rossit ARB (2004). Genetic structure of *Plasmodium falciparum* populations in the Brazilian Amazon region.. J Infect Dis.

[25] Anthony TG, Conway DJ, Cox-Singh J, Matusop A, Ratnam S (2005). Fragmented population structure of *Plasmodium falciparum* in a region of declining endemicity.. J Infect Dis.

[26] Razakandrainibe FG, Durand P, Koella JC, De Meeus T, Rousset F (2005). “Clonal” population structure of the malaria agent *Plasmodium falciparum* in high-infection regions.. Proc Natl Acad Sci USA.

[27] Mu J, Awadalla P, Duan J, McGee KM, Joy DA (2005). Recombination hotspots and population structure in *Plasmodium falciparum*.. PLOS Biology.

[28] Dondorp A, Nosten F, Stepniewska K, Day N, White N (2005). Artesunate versus quinine for treatment of severe falciparum malaria: a randomised trial.. Lancet.

[29] Cowman AF, Crabb BS (2006). Invasion of red blood cells by malaria parasites.. Cell.

[30] Anderson TJC, Su X.-Z, Bockaire M, Lagog M, Day KP (1999). Twelve microsatellite markers for characterisation of *Plasmodium falciparum* from finger prick blood samples.. Parasitology.

[31] Haubold B, Hudson RR (2000). Lian 3.0: detecting linkage disequilibrium in multilocus data.. Bioinformatics.

[32] Gardner MJ, Hall N, Fung E, White O, Berriman M (2002). Genome sequence of the human malaria parasite *Plasmodium falciparum*.. Nature.

[33] Verra F, Chokejindachai W, Weedall GD, Polley SD, Mwangi TW (2006). Contrasting signatures of selection on the *Plasmodium falciparum* erythrocyte binding antigen gene family.. Mol Biochem Parasitol.

[34] Baum J, Thomas AW, Conway DJ (2003). Evidence for diversifying selection on erythrocyte-binding antigens of *Plasmodium falciparum* and *P. vivax*.. Genetics.

[35] Neafsey DE, Schaffner SF, Volkman SK, Park D, Montgomery P (2008). Genome-wide SNP genotyping highlights the role of natural selection in *Plasmodium falciparum* population divergence.. Genome Biol.

[36] Sakihama N, Kimura M, Hirayama K, Kanda T, Na-Bangchang K (1999). Allelic recombination and linkage disequilibrium within Msp-1 of *Plasmodium falciparum*, the malignant human malaria parasite.. Gene.

[37] Anderson TJC (2004). Mapping drug resistance genes in *Plasmodium falciparum* by genome-wide association.. Curr Drug Targets Infect Disord.

[38] Pearce R, Malisa A, Kachur SP, Barnes K, Sharp B (2005). Reduced variation around drug-resistant dhfr alleles in African *Plasmodium falciparum*.. Mol Biol Evol.

[39] Wootton JC, Feng X, Ferdig MT, Cooper RA, Mu J (2002). Genetic diversity and chloroquine selective sweeps in *Plasmodium falciparum*.. Nature.

[40] WTCCC (2007). Genome-wide association study of 14,000 cases of seven common diseases and 3,000 shared controls.. Nature.

[41] Consortium M (2008). A global network for investigating the genomic epidemiology of malaria.. Nature.

[42] Tolia NH, Enemark EJ, Sim BK, Joshua-Tor L (2005). Structural basis for the EBA-175 erythrocyte invasion pathway of the malaria parasite *Plasmodium falciparum*.. Cell.

[43] Rayner JC, Tran TM, Corredor V, Huber CS, Barnwell JW (2005). Dramatic difference in diversity between *Plasmodium falciparum* and *Plasmodium vivax* reticulocyte binding-like genes.. Am J Trop Med Hyg.

[44] Gao X, Yeo KP, Aw SS, Kuss C, Iyer JK (2008). Antibodies targeting the PfRH1 binding domain inhibit invasion of *Plasmodium falciparum* merozoites.. PLoS Pathog.

[45] Mayer DCG, Mu JB, Feng X, Su XZ, Miller LH (2002). Polymorphism in a *Plasmodium falciparum* erythrocyte-binding ligand changes its receptor specificity.. J Exp Med.

[46] Mayer DCG, Mu JB, Kaneko O, Duan J, Su X.-Z (2004). Polymorphism in the *Plasmodium falciparum* erythrocyte-binding ligand JESEBL/EBA-181 alters its receptor specificity.. Proc Natl Acad Sci USA.

[47] Lobo CA, de Frazao K, Rodriguez M, Reid M, Zalis M (2004). Invasion profiles of Brazilian field isolates of *Plasmodium falciparum*: phenotypic and genotypic analyses.. Infect Immun.

[48] Jennings CV, Ahouidi AD, Zilversmit M, Bei AK, Rayner J (2007). Molecular analysis of erythrocyte invasion in *Plasmodium falciparum* isolates from Senegal.. Infect Immun.

[49] Lobo CA, Rodriguez M, Struchiner CJ, Zalis MG, Lustigman S (2006). Associations between defined polymorphic variants in the PfRH ligand family and the invasion pathways used by *P. falciparum* field isolates from Brazil.. Mol Biochem Parasitol.

[50] Gaur D, Furuya T, Mu J, Jiang LB, Su XZ (2006). Upregulation of expression of the reticulocyte homology gene 4 in the *Plasmodium falciparum* clone Dd2 is associated with a switch in the erythrocyte invasion pathway.. Mol Biochem Parasitol.

[51] Stubbs J, Simpson KM, Triglia T, Plouffe D, Tonkin CJ (2005). Molecular mechanism for switching of *P. falciparum* invasion pathways into human erythrocytes.. Science.

[52] Hayton K, Gaur D, Liu A, Takahashi J, Henschen B (2008). Erythrocyte binding protein PfRH5 polymorphisms determine species-specific pathways of *Plasmodium falciparum* invasion.. Cell Host Microbe.

