# Supplementary material for: Case-Control Approach to Identify Plasmodium falciparum Polymorphisms Associated with Severe Malaria
Source: PLoS One. 2009 May 6;4(5):e5454. doi: 10.1371/journal.pone.0005454 (PMC2674215; doi:10.1371/journal.pone.0005454)
Supplement: Table S1 — (0.10 MB DOC) [file pone.0005454.s001.doc]

Supplementary Table 1 A. Primer sequences for known microsatellites on *chr 2*

| For each locus the second and third primers were used in the primary reaction and the first and second primers were used in the secondary reaction. All primers are listed 5’ → 3’. The sequence of primer is based on genetic map in GenBank (*Su et a*l. 1999). <http://www.ncbi.nlm.nih.gov/Malaria/Mapsmarkers/PfGMap/pfgmap_2.html> |
| --- |

| Name | Label |  | Accession No. | Sequence |
| --- | --- | --- | --- | --- |
| C2M32-R | NED |  | G37994 | GGCCTTATAAAAATTATACG |
| C2M32-F |  |  |  | TTTTATAGGGGGATTTAGTA |
| C2M32-3(R ) |  |  |  | AAAATCGTTAACAGGAATATA |
|  |  |  |  |  |
| IMP-F | HEX |  | G37800 | TCGAATGTATATATGGCA |
| IMP-R |  |  |  | TTATCTTTTGCCATATTC |
| IMP-3(F) |  |  |  | ACATAATCAATGTGGTGAC |
|  |  |  |  |  |
| C2M33-F | FAM |  | G37886 | CATTGCAAAATATATATTCTCC |
| C2M33-R |  |  |  | GTGATTTGTACAATGTACATA |
| C2M33-3(F) |  |  |  | GCGTAAATAACACATCTGC |
|  |  |  |  |  |
| SERP2-F | NED |  | G37768 | AATTGGAAGCTAAGGTAT |
| SERP2-R |  |  |  | CAATAAAGAGGAATATACA |
| SERP2-3(F) |  |  |  | TTTCAATGTTAAAATTACCAC |
|  |  |  |  |  |
|  |  |  |  |  |
| C2M34-F | FAM |  | G37995 | TCCCTTTTAAAATAGAAGAAA |
| C2M34-R |  |  |  | GATTATATGAAAGGATACATG |
| C2M34-3(F) |  |  |  | CTTTGTAAATTAGAACATATC |
|  |  |  |  |  |
| C2M29-F | HEX |  | G37891 | GTGAATAACGGAAAAGGATA |
| C2M29-R |  |  |  | AAGATCAAATACCAGGTGA |
| C2M29-3(F) |  |  |  | AAACAATCAGAAGCGATGG |
|  |  |  |  |  |
| C2M28-F | HEX |  | G37890 | ATGGGAAATAAAAATTGAATG |
| C2M28-R |  |  |  | GTGTTCAAATTATTAGTTACG |
| C2M28-3(F) |  |  |  | CTATGTTCATATATGGAATG |
|  |  |  |  |  |
| C2M27-F | NED |  | G37889 | CTTTTAATCACTACCATGTTG |
| C2M27-R |  |  |  | ATAATTTAATTGAGGATACCT |
| C2M27-3(F) |  |  |  | TTTCTATTTTTACAATTACGTG |
|  |  |  |  |  |
| B7M51-F | FAM |  | G42713 | AAATATAAATCTTCTTCTTCTTTTTT |
| B7M51-R |  |  |  | TAGAGAAATAAATATATCCAT |
| B7M51-3(F) |  |  |  | GTTCATATAAGTTTGCTAGG |

Primary and secondary round reactions were multiplexed in the following combinations: *C2M34*, *C2M28* and *SERP2*; *B7M51*, *IMP* and *C2M27*; *C2M33*, *C2M29* and *C2M32*.

Supplementary Table 1 B. Primer sequences for new microsatellites on *chr 2*

| For each locus the second and third primers were used in the primary reaction and the first and second primers were used in the secondary reaction, except *M3508* and *M3596* that used the same primer, M3475-3(F) and M3762-3(R) in the primary reaction, then the first and second primers, and third and fourth primers were used in the secondary reaction. All primers are listed 5’ → 3’. | | | | | |
| --- | --- | --- | --- | --- | --- |
|  | Name |  | Label | Sequence |  |
|  | M404-F |  | NED | TAAAGTTGAACAAGTACAC |  |
|  | M404-R |  |  | TATTACCTTAAAGAGGTGT |  |
|  | M404-3(F) |  |  | TAGTCTTCTGTTAATACAG |  |
|  |  |  |  |  |  |
|  | M2818-R |  | NED | AAAATTATACAAATCAAGTATA |  |
|  | M2818-F |  |  | TTTATTTGGTATCTATAGATA |  |
|  | M2818-3(R) |  |  | GGTTGTAGATATAAAGAATTA |  |
|  |  |  |  |  |  |
|  | M3140-F |  | HEX | TTTTAATATAAGATATACATGA |  |
|  | M3140-R |  |  | GCATGCTTTAATAGGTAAG |  |
|  | M3140-3(F) |  |  | CGAAAACTTAAAGGGATGT |  |
|  |  |  |  |  |  |
|  | M3475-3(F) |  |  | GCCTTTAATAACTATTTAGA |  |
|  | M3508-R |  | NED | GTGACCATTCATAAGTCA |  |
|  | M3596-F |  | FAM | GGTCACACACACATGTAT |  |
|  | M3762-3(R) |  |  | GTCATTCATTAATCTTCTGA |  |
|  |  |  |  |  |  |
|  | M4252-R |  | HEX | CTCATCATCTACATTGTC |  |
|  | M4252-F |  |  | AAATATAGTACACACACGA |  |
|  | M4252-3(R) |  |  | CTATAATGTGGCACCTCA |  |
|  |  |  |  |  |  |
|  | M6554-F |  | FAM | GCCATATTCATATAATTCTC |  |
|  | M6554-R |  |  | CGTATACATAATCAATGTG |  |
|  | M6554-3(F) |  |  | AGCAACACTTGTTTTATCT |  |
|  |  |  |  |  |  |
|  | M6892-R |  | HEX | CGTACTTGAAGAAATATGG |  |
|  | M6892-F |  |  | TTAGTAGTAATAAATTGTACA |  |
|  | M6892-3(R) |  |  | CATAATGCGTACTTGAAG |  |
|  |  |  |  |  |  |
|  | M7100-F |  | HEX | CCCAACTTGCAGTGTCT |  |
|  | M7100-R |  |  | TCCATGTCTTAAGGTATC |  |
|  | M7100-3(F) |  |  | GCGTACATGTTATAAGGT |  |
|  |  |  |  |  |  |
|  | M9999-R |  | FAM | GCTGATCTTATTTGTAAAG |  |
|  | M9999-F |  |  | AAAATTCGAACATGTGATTA |  |
|  | M9999-3(R) |  |  | CAGTGATAGTTACATTTTAC |  |

Primary and secondary round reactions were multiplexed in the following combinations: *M2818*, *M3140* and *M3596*; *M3508*, *M4252* and *M6554*; *M404*, *M6892*, *M7100* and *M9999*.
