# Supplementary material for: Case-Control Approach to Identify Plasmodium falciparum Polymorphisms Associated with Severe Malaria
Source: PLoS One. 2009 May 6;4(5):e5454. doi: 10.1371/journal.pone.0005454 (PMC2674215; doi:10.1371/journal.pone.0005454)
Supplement: Table S2 — (0.15 MB DOC) [file pone.0005454.s002.doc]

| **Supplementary Table 2. Sequence specific oligonucleotide probes (SSOP) for typing *eba175*, *eba140* and *eba181* polymorphisms** |
| --- |

| Codon (nt) | Amino acid polymorphisms (SNPs) | Oligonucleotide probes | Hybe temp (°C) | Wash temp (°C) |
| --- | --- | --- | --- | --- |
| ***eba175*** |  |  |  |  |
| 157 (470) | **N**/**S** (A**A**T 🡪 A**G**T) | 157***N** TTAAGTA**A**TTGTAGGGAA  157***S** TTAAGTA**G**TTGTAGGGAA | 53 | 57 |
| 274 (820) | **K**/**E** (**A**AA 🡪 **G**AA) | 274***K** CATGGG**A**AAATAAGTGAA  274***E** CATGGG**G**AAATAAGTGAA | 53 | 58 |
| 279 (835) | **E**/**K** (**G**AA 🡪 **A**AA) | 279***E** AGTGAACAT**G**AAATTAAA  279***K** AGTGAACAT**A**AAATTAAA | 55 | 63 |
| 286 (856) | **E**/**K** (**G**AA 🡪 **A**AA) | 286***E** TTTAGAAAA**G**AATGGTGG  286***K** TTTAGAAAA**A**AATGGTGG | 55 | 63 |
| 336 (1006) | **D**/**Y** (**G**AT 🡪 **T**AT) | 336***D** CTTGAAAGA**G**ATAATAGA  336***Y** CTTGAAAGA**T**ATAATAGA | 55 | 61 |
| 388/390 (1164/1168) | **N**/**K** (AA**T** 🡪 AA**A**)  **S**/**P** (**T**CA 🡪 **C**CA) | 388/390***NS** ACTCAAAA**T**GTT**T**CAAAG  388/390***KS** ACTCAAAA**A**GTT**T**CAAAG  388/390***KP** ACTCAAAA**A**GTT**C**CAAAG | 55 | 63 |
| 401-405 (1201-1215) | **IS**/**de**l (**ATTTCA** 🡪 **del** )  403 **E**/**K** (**G**AA 🡪 **A**AA)  404 **N**/**K** (AA**C** 🡪 AA**A**)  405 **K**/**M** (A**A**G 🡪 A**T**G) | 401-5***ISENK** **ATTTCAG**AAAA**C**A**A**GAAT  401-5***ISKNK** **ATTTCAA**AAAA**C**A**A**GAAT  401-5*--**KKM** AAA**------A**AAAA**A**A**T**GAATGAT | 55 | 62.5 |
| 481 (1442) | **I**/**K** (A**T**A 🡪 A**A**A) | 481***I** CCTTATA**T**ATTATCCACT  481***K** CCTTATA**A**ATTATCCACT | 54.5 | 63.5 |
| 577 (1731) | **K**/**N** (AA**A** 🡪 AA**T**) | 577***K** AATTCAAA**A**TATGTTCAC  577***N** AATTCAAA**T**TATGTTCAC | 53 | 56 |
| 584 (1750) | **K**/**E**/**Q** (**A**AA 🡪 **G**AA 🡪 **C**AA) | 584***K** AGGAATAAA**A**AAAATGAT  584***E** AGGAATAAA**G**AAAATGAT  584***Q** AGGAATAAA**C**AAAATGAT | 54.5 | 63 |
| 592 (1775) | **E**/**A** (G**A**G🡪 G**C**G) | 592***E** TTTCGTGATG**A**GTGGTGG  592***A** TTTCGTGATG**C**GTGGTGG | 54.5 | 63 |
| 664 (1990) | **S**/**R** (**A**GT 🡪 **C**GT) | 664***S** AATTGTAAA**A**GTAAATGT  664***R** AATTGTAAA**C**GTAAATGT | 54.5 | 61.5 (S)  62.5 (R) |
| 716 (2146) | **E**/**K** (**G**AA 🡪 **A**AA) | 716***E** AAATACTCG**G**AAAAATGT  716***K** AAATACTCG**A**AAAAATGT | 54.5 | 63.5 |
| ***eba140*** |  |  |  |  |
|  |  |  |  |  |
| 185 (553) | **V/I** (**G**TT/**A**TT) | 185***V** CTATGT**G**TTGCAAATTTT  185***I** CTATGT**A**TTGCAAATTTT | 54.5 | 58.5 |
| 239 (716) | **S/N** (A**G**T/A**A**T) | 239***S** TTTAGAA**G**TTCATTTATA  239***N** TTTAGAA**A**TTCATTTATA | 53 | 56 |
| 261 (782) | **K/T/R** (A**A**G/A**C**G/A**G**G) | 261***K** ATTAATA**A**GAAGTTCTCC  261***T** ATTAATA**C**GAAGTTCTCC  261***R** ATTAATA**G**GAAGTTCTCC | 54.5 | 59.5 |
| 285 (853,855) | **K1/K2/N/E****  (**A**AA/AA**G**/AA**C/G**AA) | 285***K1** TGGGAA**A**AAAATAAAGCA  285***K2** TGGGAAAA**G**AATAAAGCA  285***N** TGGGAAAA**C**AATAAAGCA  285***E** TGGGAA**G**AAAATAAAGCA | 54.5 | 59.2 |
| ***eba181*** |  |  |  |  |
|  |  |  |  |  |
| 359 (1076) | **R/K** (A**G**G/A**A**G) | 359***R** ATATTTA**G**GAGAAAAATA  359***K** ATATTTA**A**GAGAAAAATA | 53 | 56 |
| 363-4 (1088/1092) | **VQ/DQ/VH****  (G**T**T CA**A/**G**A**T CA**A/**  G**T**T CA**C**) | 363-4***VQ** AAAATAG**T**TCA**A**CCTGAT  363-4***DQ** AAAATAG**A**TCA**A**CCTGAT  363-4***VH** AAAATAG**T**TCA**C**CCTGAT | 54.5 | 59 |
| 414 (1241) | **I/N** (A**T**T/A**A**T) | 414***I** AAAAATA**T**TGATATTTGT  414***N** AAAAATA**A**TGATATTTGT | 54.5 | 59 |
| 443 (1327) | **Q/K** (**C**AA/**A**AA) | 443***Q** CCATGG**C**AATGTGATAAA  443***K** CCATGG**A**AATGTGATAAA | 54.5 | 59.2 |
| 637 (1911) | **N/K** (AA**T/**AA**A**) | 637***N** AAATTAAA**T**GATAAATGT  637***K** AAATTAAA**A**GATAAATGT | 54.5 | 59.2 (N)  58 (K) |
|  |  |  |  |  |
|  |  |  |  |  |

** The allele result of Camp genotyped by SSOP is *eba140*-285E not K2(AA**G**) as in Genbank/EMBL/DDBJ (AF507990) and confirmed on all replicate membranes.

** VH only seen in D10 clone (AY495335) and not available in this study for the positive control.

**PCR Amplification**: Two fragments of the *eba-175* gene were amplified separately by nested PCR, representing codons 145- 652 (primers: FWD 5´- ggaagaaatacttcatctaataacg - 3´, REV 5´ - gcattcaaccttcagagtc - 3´) and heminested PCR at codon 467- 780 (FWD 5´- gttgatacaaacacaaaggtg - 3´, REV 5´ - gagatagatggagtttccgttctg - 3´) from a first round PCR of codon 125- 780 (FWD 5´- cctattaacgctgtacgtgtgtc - 3´, REV 5´ - gagatagatggagtttccgttctg - 3´); codon numbers based on the CAMP sequence; X52524. Fragments of the *eba-140* and *eba-181* genes were amplified representing codon 163- 298 (FWD 5´- gatatgtcgagaaaacagtgaga - 3´, REV 5´ - gtggaatcacatgatagtaaatca - 3´) of the *eba-140* Dd2 sequence; AF332918 and 261- 778 (FWD 5´- actatggaaaactatgatacagcc - 3´, REV 5´ - cgatttctttattaccacttgcg - 3´) of the *eba-181* Dd2 sequence; AB080796. PCR reactions were performed using optimised polymerase and dNTP mix in a ready-to-go format (BioMix Red, Bioline Ltd.) with a total volume of 40 µL for *eba-175* and 25 µL for *eba-140* and *eba-181* amplification in each well of 96-well plates*.*

**Sequence-specific oligonucleotide probing (SSOP):** After amplification, products were denatured for 2 min at 94ºC and 1.5 µL aliquots were dotted in 96-sample arrays on replicate nylon membranes (MagnaGraph™ or Sigma™), and cross-linked by ultra-violet light prior to hybridisation assays. Allelic probes were labelled with digoxigenin in separate tubes simultaneously, under identical conditions using the Boehringer Mannheim 3’-end labelling kit, and labelled probes were used for hybridisation at a final concentration of 2 nM in TMAC hybridisation buffer (3 M Tetramethylammonium chloride, 50 mM Tris pH 8.0, 2 mM EDTA pH 8.0, 0.1 % SDS). After blocking membranes for 30 mins in 1 % milk powder, hybridisation was performed at for 90 mins at 53-55^o^C (temperature for each probe specified in table), followed by 2 x 10 min low-stringency washes at room temperature (in 2 x SSPE, 0.1 % SDS), and 2 x 10 min high-stringency washes at 55-63.5^o^C (temperature for each probe as specified in the Table) in TMAC buffer. Detection of hybridised dig-labelled probes on the membranes was performed by probing with anti-digoxigenin Fab fragment conjugated with alkaline phosphatase (Boehringer Mannheim) followed by detection using CSPD substrate and exposure on Hyperfilm-ECL. These conditions allowed clear and accurate discrimination between alleles, including those differing at a single nucleotide position, as confirmed by the inclusion of allele-specific controls to be typed in each assay.
