# Supplementary material for: Case-Control Approach to Identify Plasmodium falciparum Polymorphisms Associated with Severe Malaria
Source: PLoS One. 2009 May 6;4(5):e5454. doi: 10.1371/journal.pone.0005454 (PMC2674215; doi:10.1371/journal.pone.0005454)
Supplement: Table S3 — (0.14 MB DOC) [file pone.0005454.s003.doc]

Supplementary Table 3. Oligonucleotide sequences for typing simple sequence repeat polymorphisms in *Rh* genes

All primers are listed 5’ → 3’. For each locus the first and second primers were used in the primary reaction and the second and third primers were used in the secondary reaction. The third primer is the dye-labeled primer.

| Name with position | label | Repeat | Oligonucleotide sequence |
| --- | --- | --- | --- |
| Rh1_int -3R |  | (TAAACATATA)n | agaataactttcctgttcatgg |
| Rh1_int -F |  |  | gcatatattaatttacttagcagg |
| Rh1_int -R | 6-FAM |  | gttcatggctaaattctgggg |
|  |  |  |  |
| Rh1_667-3F |  | (AAAAAACAAACA)n | atgaagcacaattagacattacc |
| Rh1_667-R |  |  | aatcgtctcattattttgtattag |
| Rh1_667-F | VIC |  | acattaccctcctcgatgac |
|  |  |  |  |
| Rh1_1309-3R |  | (ATAAACGATATTGATGAA)n | cacgtgtatcatcagcatgg |
| Rh1_1309-F |  |  | cgatgcggatgatacaaacg |
| Rh1_1309-R | PET |  | catcagcatggtatatatcgtc |
|  |  |  |  |
| Rh1_2856-3R |  | (CATAAT)n + (CAAAAT)n | ttatctttactttgttgctcatg |
| Rh1_2856-F |  |  | aatatcaaaccacatcatgttc |
| Rh1_2856-R | FAM |  | gtgtgtagatatatcttgttcc |
|  |  |  |  |
| Rh2ab_int-3F |  | (AT)n | tgtatttttagatctttacaaagg |
| Rh2ab_int-R |  |  | aactgaagatgctccatggg |
| Rh2ab_int-F | 6-FAM |  | tctataatatttactactaaagcg |
|  |  |  |  |
| Rh2ab_747-3F |  | (AAT)n | gaactatctcattttttagacac |
| Rh2ab_747-R |  |  | gacttaatagatgacttaattcag |
| Rh2ab_747-F | VIC |  | aatacatcaaataatgaatgtgct |
|  |  |  |  |
| Rh4_int-3R |  | (TA)n | gttcttagaatctgcttctgg |
| Rh4_int-F |  |  | gatcataagtaaaacgacacac |
| Rh4_int-R | 6FAM |  | ggaattgcgtcatttccttgg |
|  |  |  |  |
| Rh4_820-3R |  | (AAT)n | aagatgtgattataatcaccgg |
| Rh4_820-F |  |  | tagggacataaaatatatagtcc |
| Rh4_820-R | NED |  | aatcaccggtttgattattacc |
|  |  |  |  |
| Rh4_1133-3R |  | In-del | tacatcctttttgatttgtatgc |
| Rh4_1133-F |  | GACCATAATGAT | atacaaaatcaaaggattcattac |
| Rh4_1133-R | PET |  | cctttttgatttgtatgcattcg |
|  |  |  |  |
| Rh4_1754-3F |  | In-del | tttagtgaagcagataatgcac |
| Rh4_1754-R |  | AATGAA | atatgtcattaaaatcttcattttc |
| Rh4_1754-F | VIC |  | aaagaagaatatctacctgtcta |
|  |  |  |  |

The number in the primer name corresponds to the codon position at the middle point of the amplified fragment. Primers that amplify introns are labelled ‘int’.
