# Supplementary figures and images for: Case-Control Approach to Identify Plasmodium falciparum Polymorphisms Associated with Severe Malaria
Source: PLoS One. 2009 May 6;4(5):e5454. doi: 10.1371/journal.pone.0005454 (PMC2674215; doi:10.1371/journal.pone.0005454)

## Slide 1
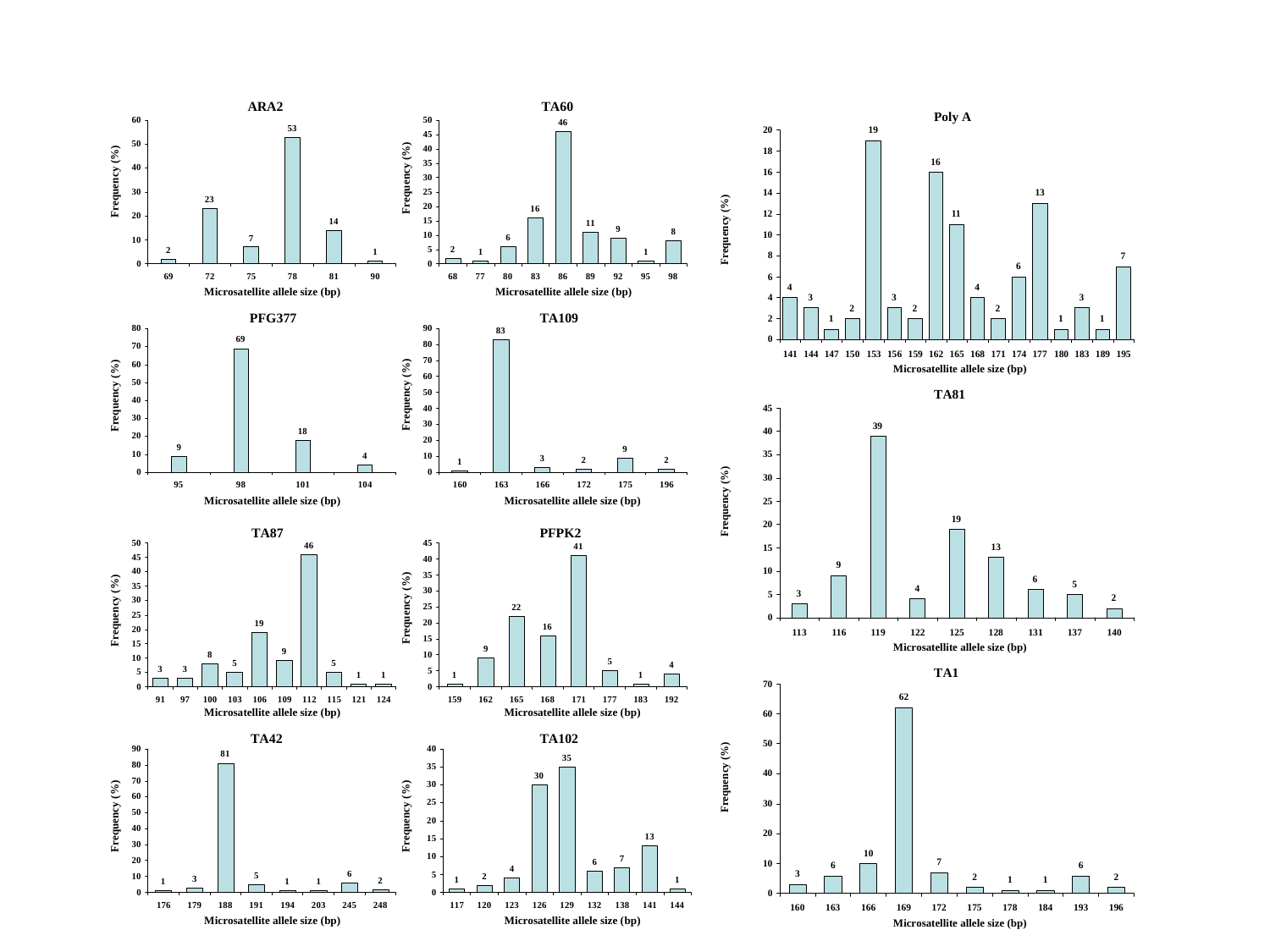

Supplement: Figure S1 — Allele frequencies at 11 widely separated microsatellite loci in 100 Plasmodium falciparum isolates in Thailand (0.06 MB PPT) [file pone.0005454.s004.ppt]

## Slide 1
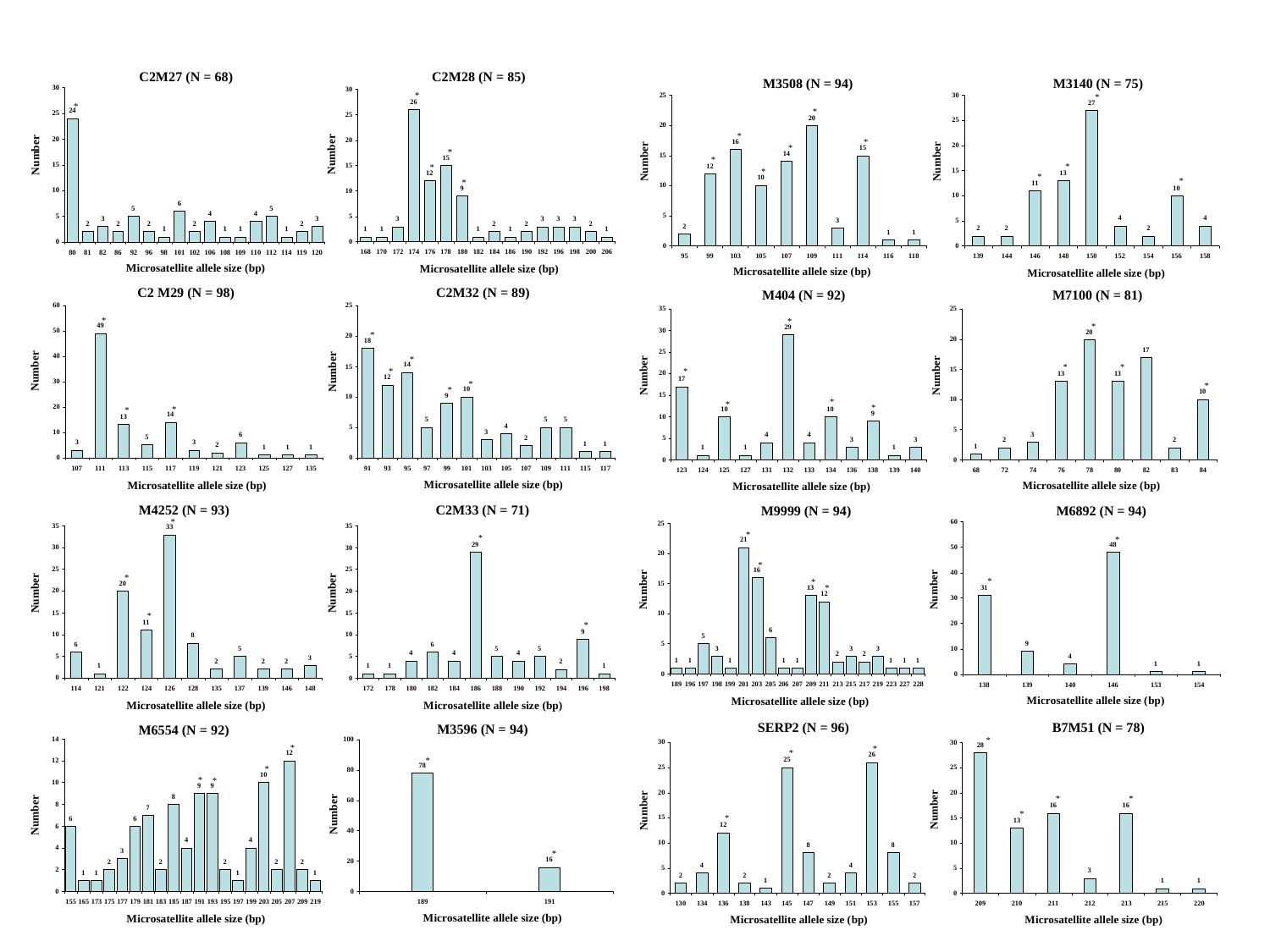

Supplement: Figure S2 — Allele frequencies at 16 microsatellite loci in a 140 kb region of Chromosome 2 in a sample of 100 Plasmodium falciparum isolates in Thailand (asterisks show alleles used in LD analyses) (0.08 MB PPT) [file pone.0005454.s005.ppt]
